# Supplementary material for: Striking Differences in Platelet Distribution between Advanced-Platelet-Rich Fibrin and Concentrated Growth Factors: Effects of Silica-Containing Plastic Tubes
Source: J Funct Biomater. 2019 Sep 17;10(3):43. doi: 10.3390/jfb10030043 (PMC6787607; doi:10.3390/jfb10030043)
Supplement: Supplementary file 1 [file jfb-10-00043-s001.pdf]

Journal of Functional Biomaterials

MDPI

a) Plain glass tube (A-PRF+) (low-speed)

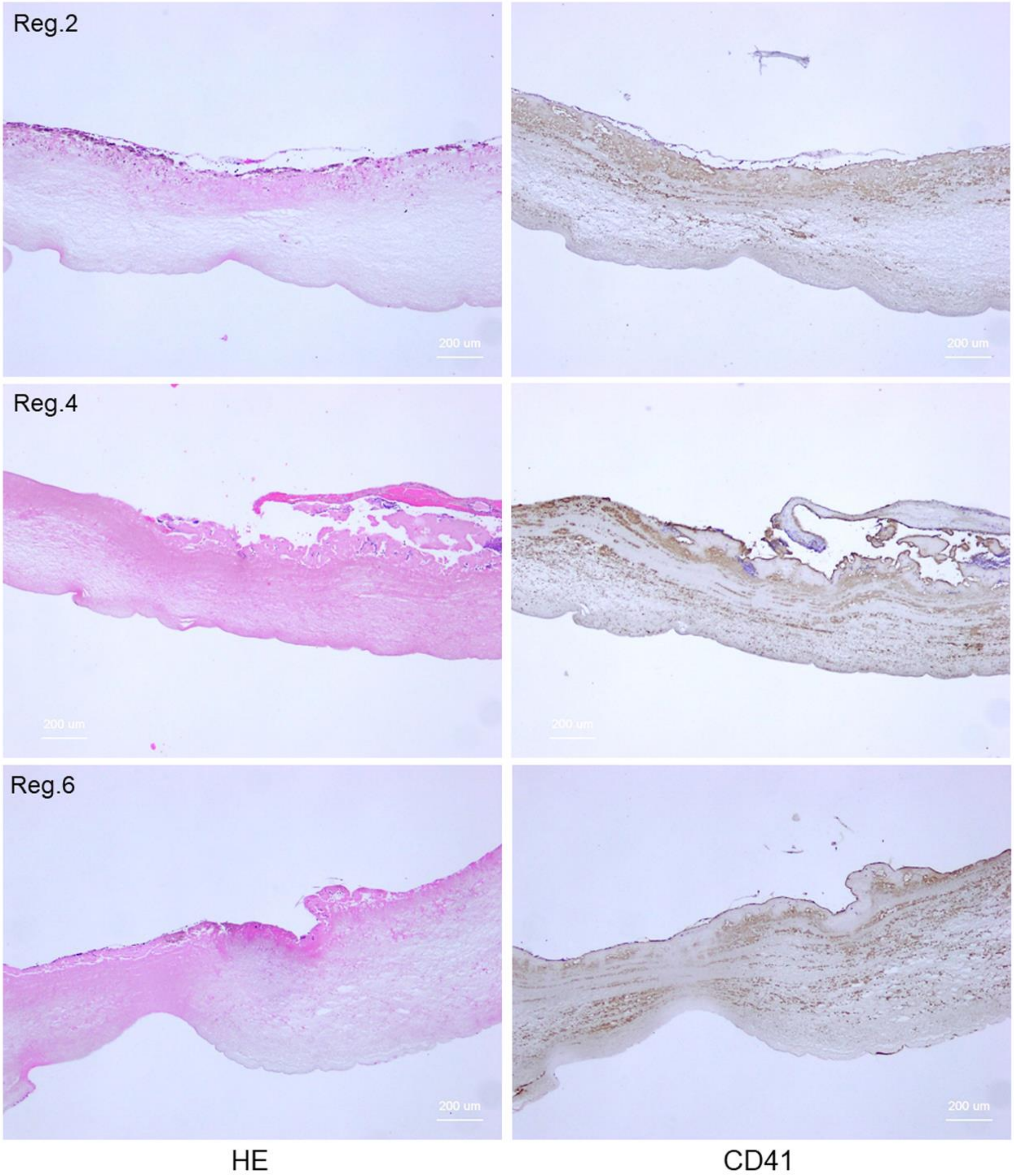

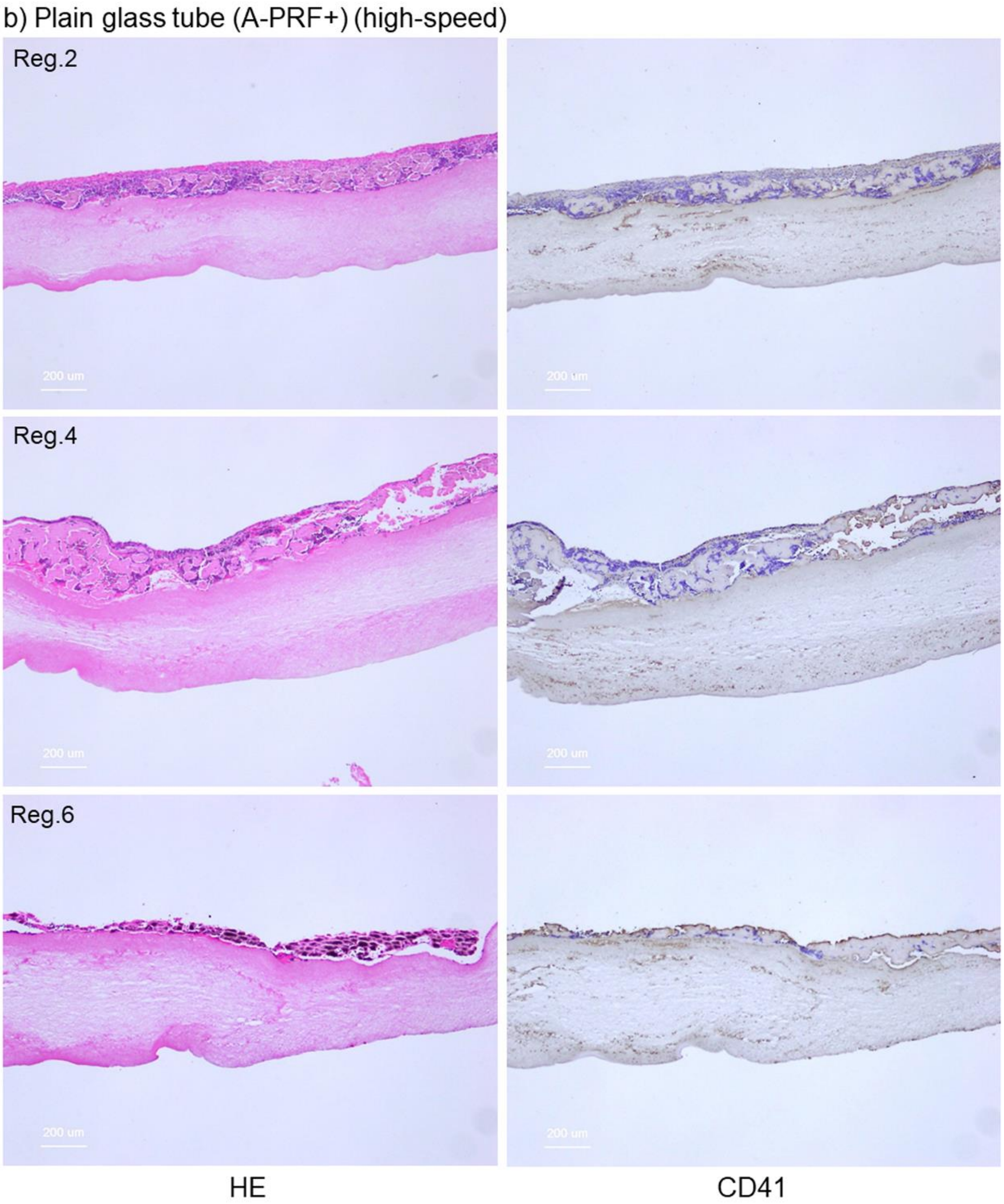

Figure S1. Platelet distribution in PRF matrix prepared using glass A-PRF+ tubes by low- (**a**: A-PRF protocol) and high-speed centrifugation (**b**: CGF protocol). Sections from regions (Reg.) 2, 4, and 6, stained with HE (left panels) or using anti-CD41 antibody (right panels) are shown. CD41⁺ platelets are stained dark brown.

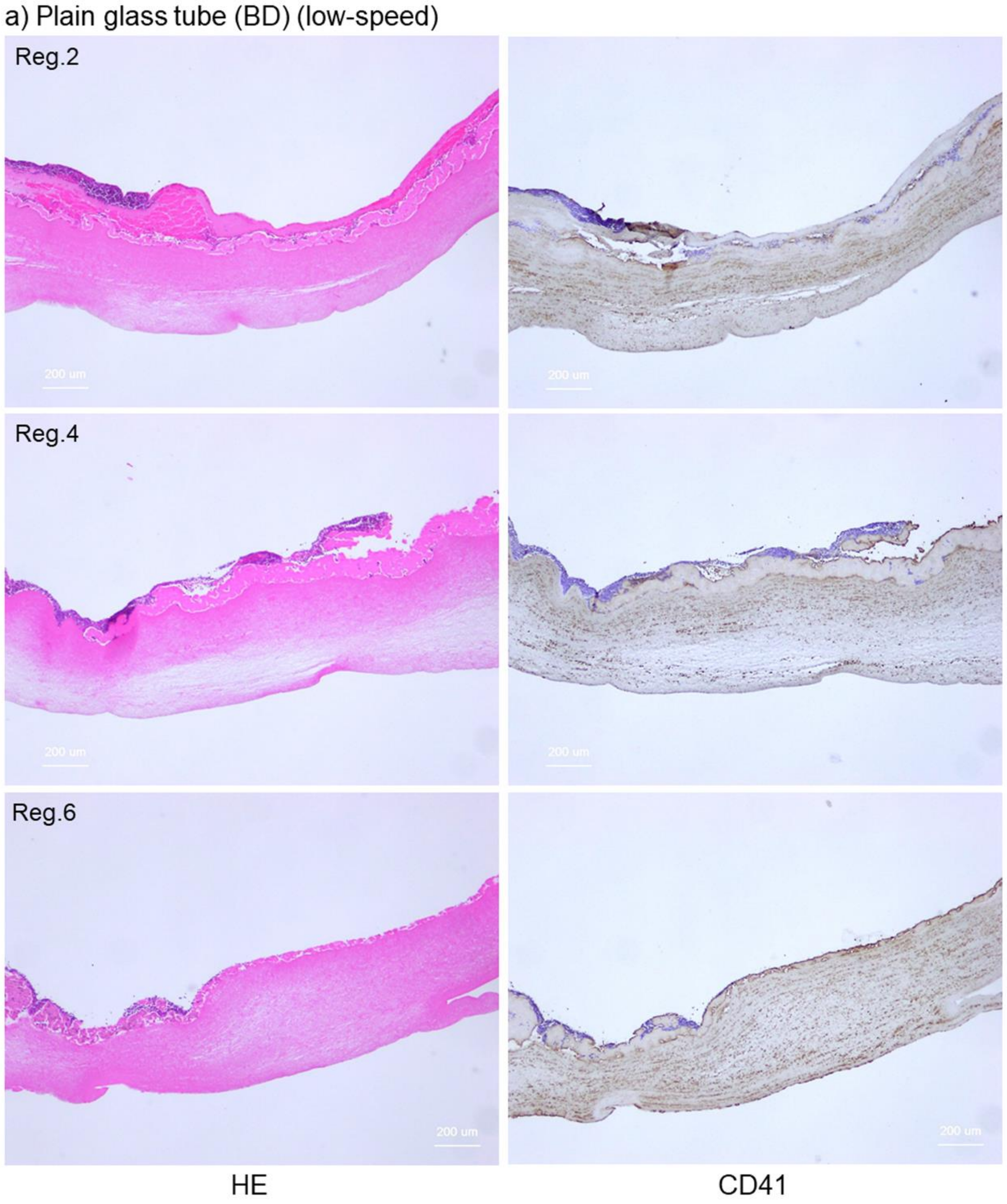
a) Plain glass tube (BD) (low-speed)
Reg.2
Reg.4
Reg.6
200 um
HE
CD41

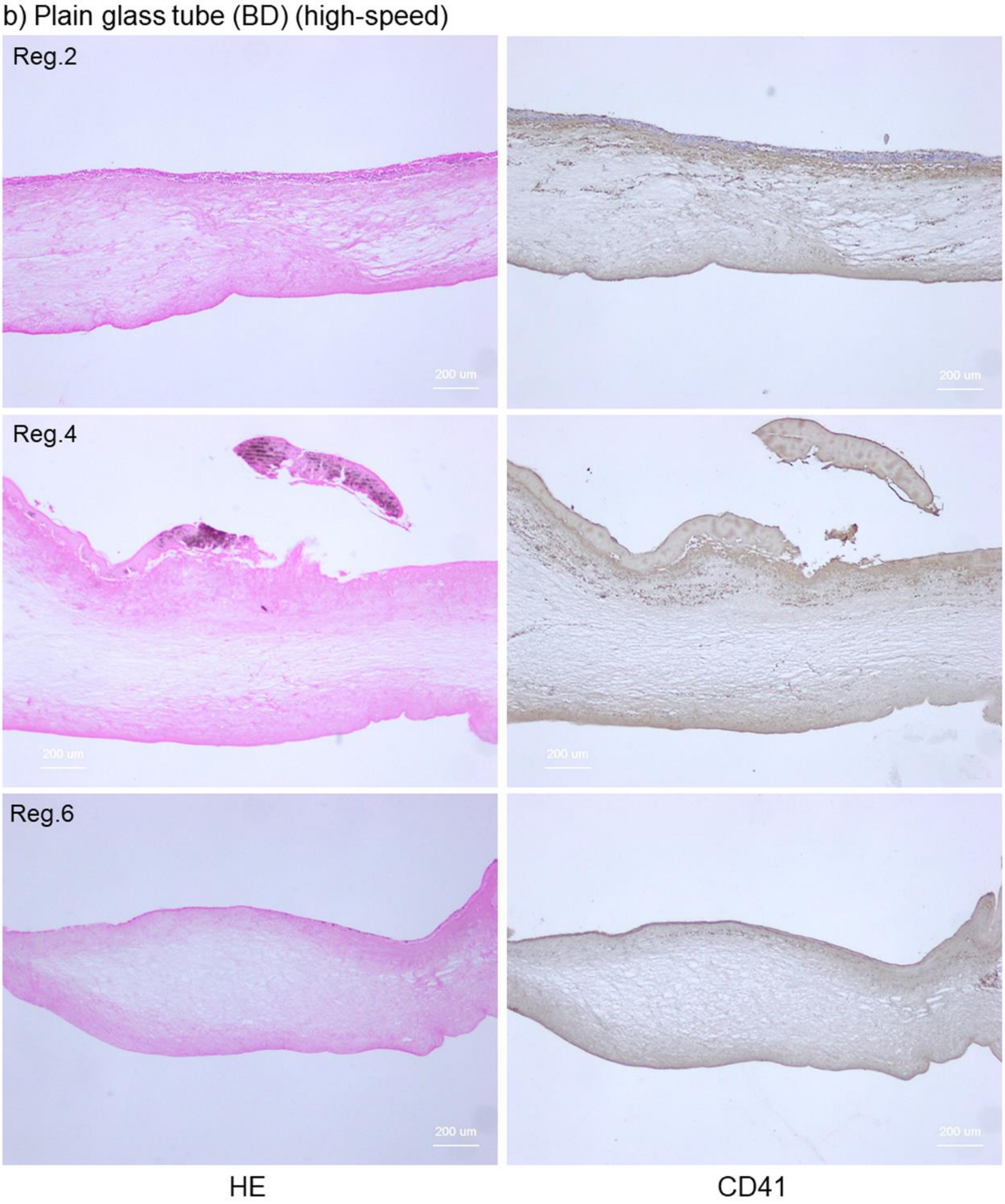

Figure S2. Platelet distribution in PRF matrix prepared using glass Vacutainer tubes by low- (**a**: A-PRF protocol) and high-speed centrifugation (**b**: CGF protocol). Sections from regions (Reg.) 2, 4, and 6, stained with HE (left panels) or using anti-CD41 antibody (right panels) are shown. $CD41^{+}$ platelets are stained dark brown.

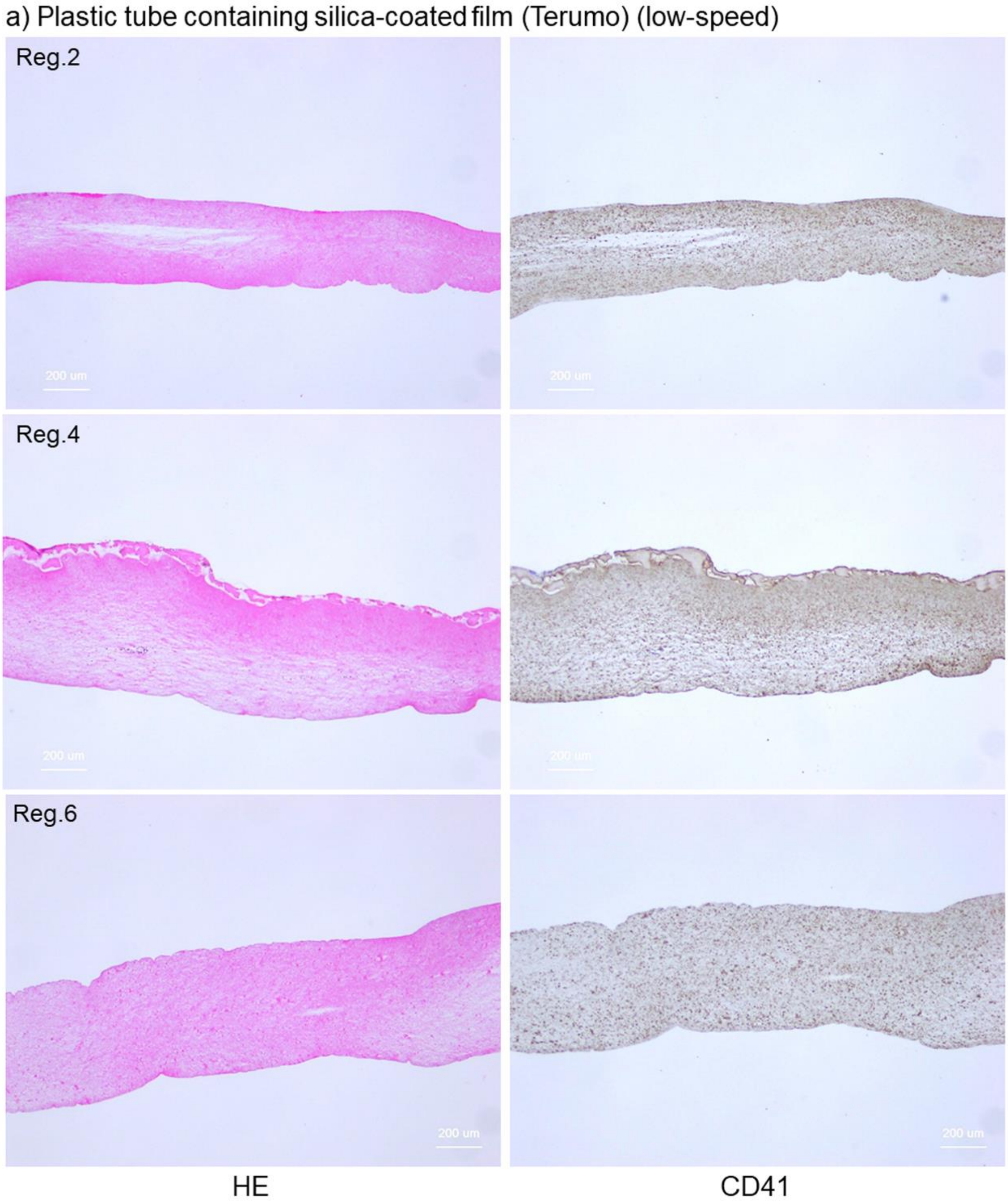
a) Plastic tube containing silica-coated film (Terumo) (low-speed)
Reg.2
Reg.4
Reg.6
200 um
HE
CD41

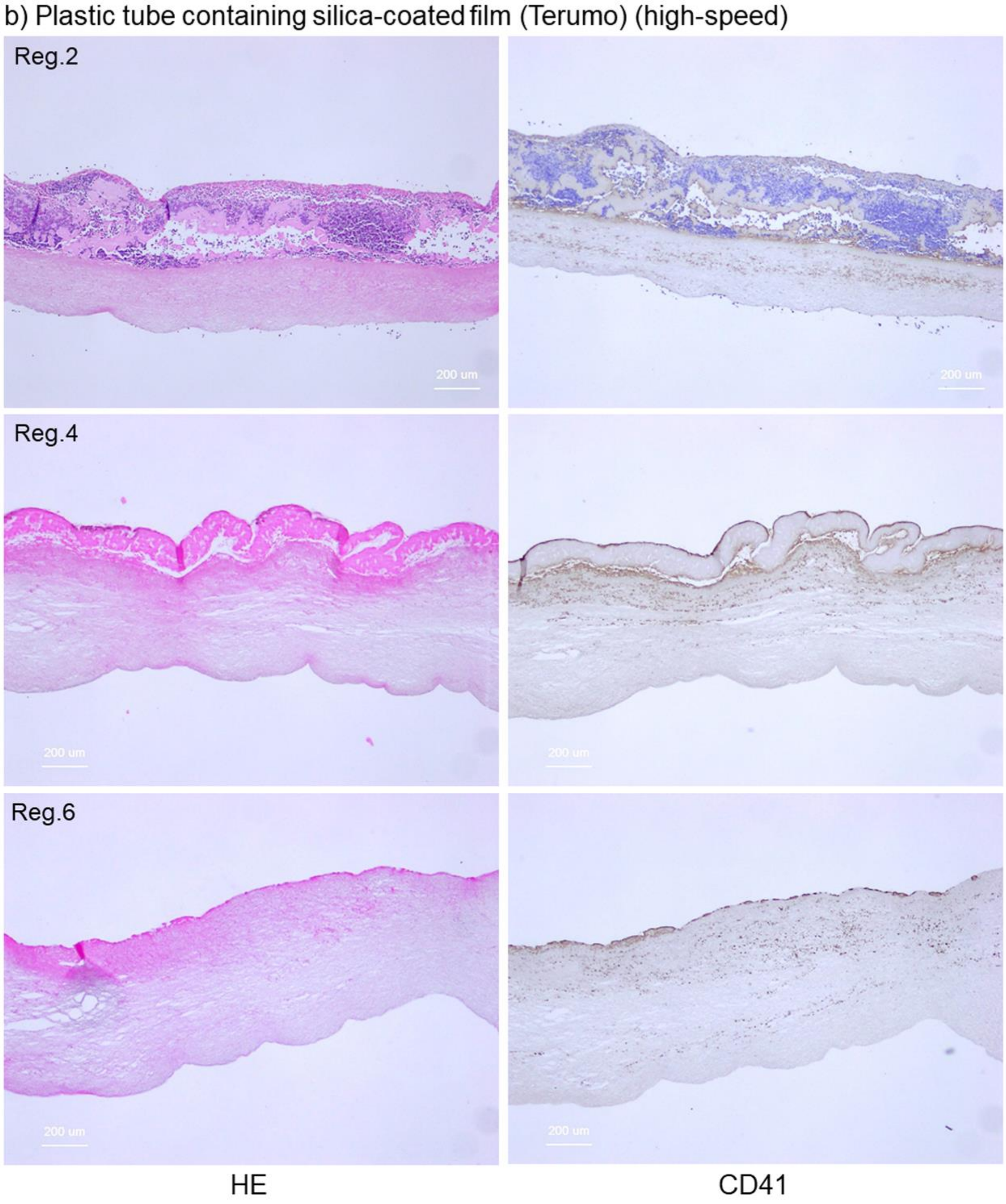

Figure S3. Platelet distribution in PRF matrix prepared using plastic tubes containing silica-coated film (Venoject II) by low- (**a**: A-PRF protocol) and high-speed centrifugation (**b**: CGF protocol). Sections from regions (Reg.) 2, 4, and 6, stained with HE (left panels) or anti-CD41 antibody (right panels) are shown. CD41^{+} platelets are stained dark brown.

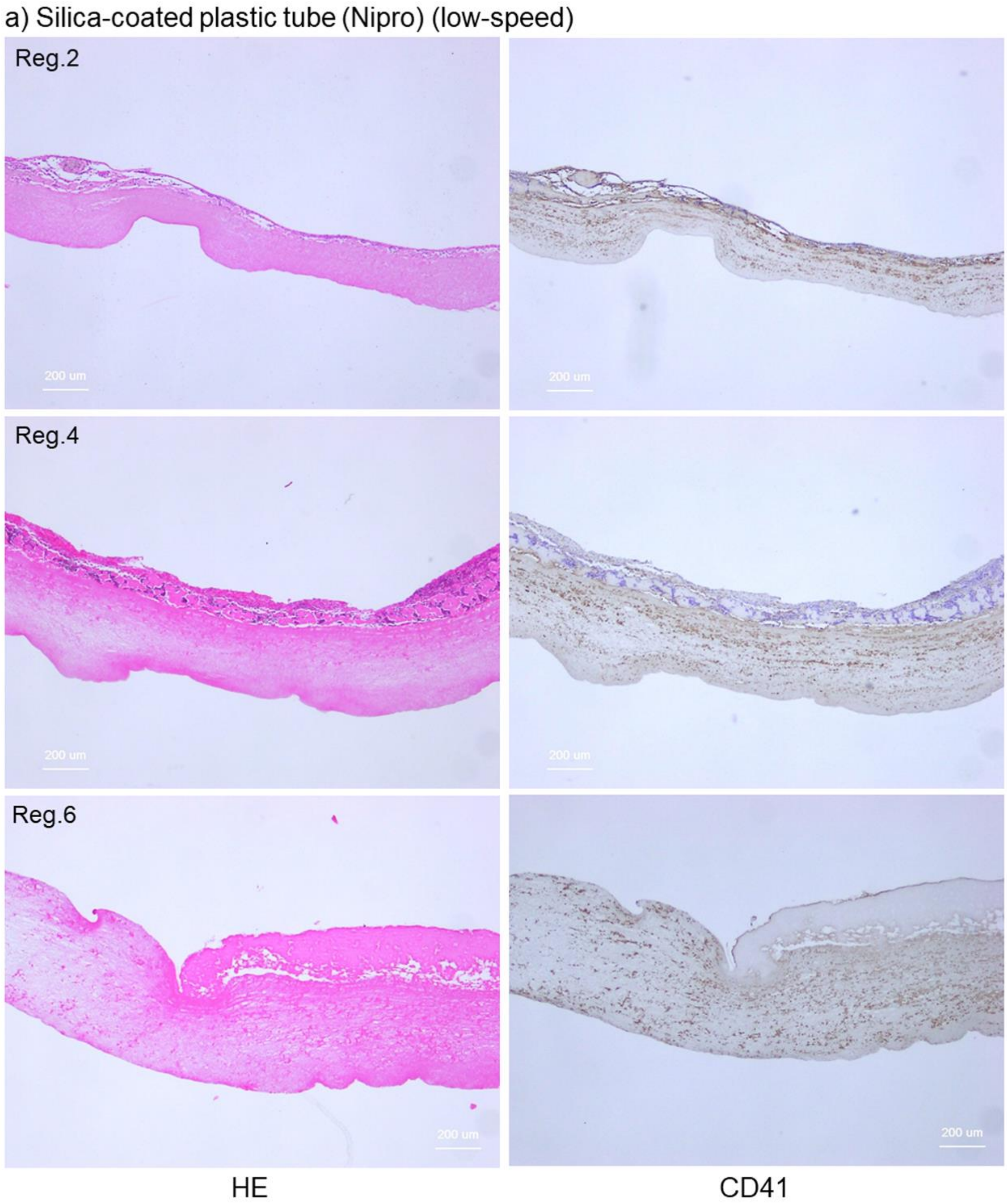
a) Silica-coated plastic tube (Nipro) (low-speed)
Reg.2
200 um
200 um
Reg.4
200 um
200 um
Reg.6
200 um
200 um
HE
CD41

b) Silica-coated plastic tube (Nipro) (high-speed)

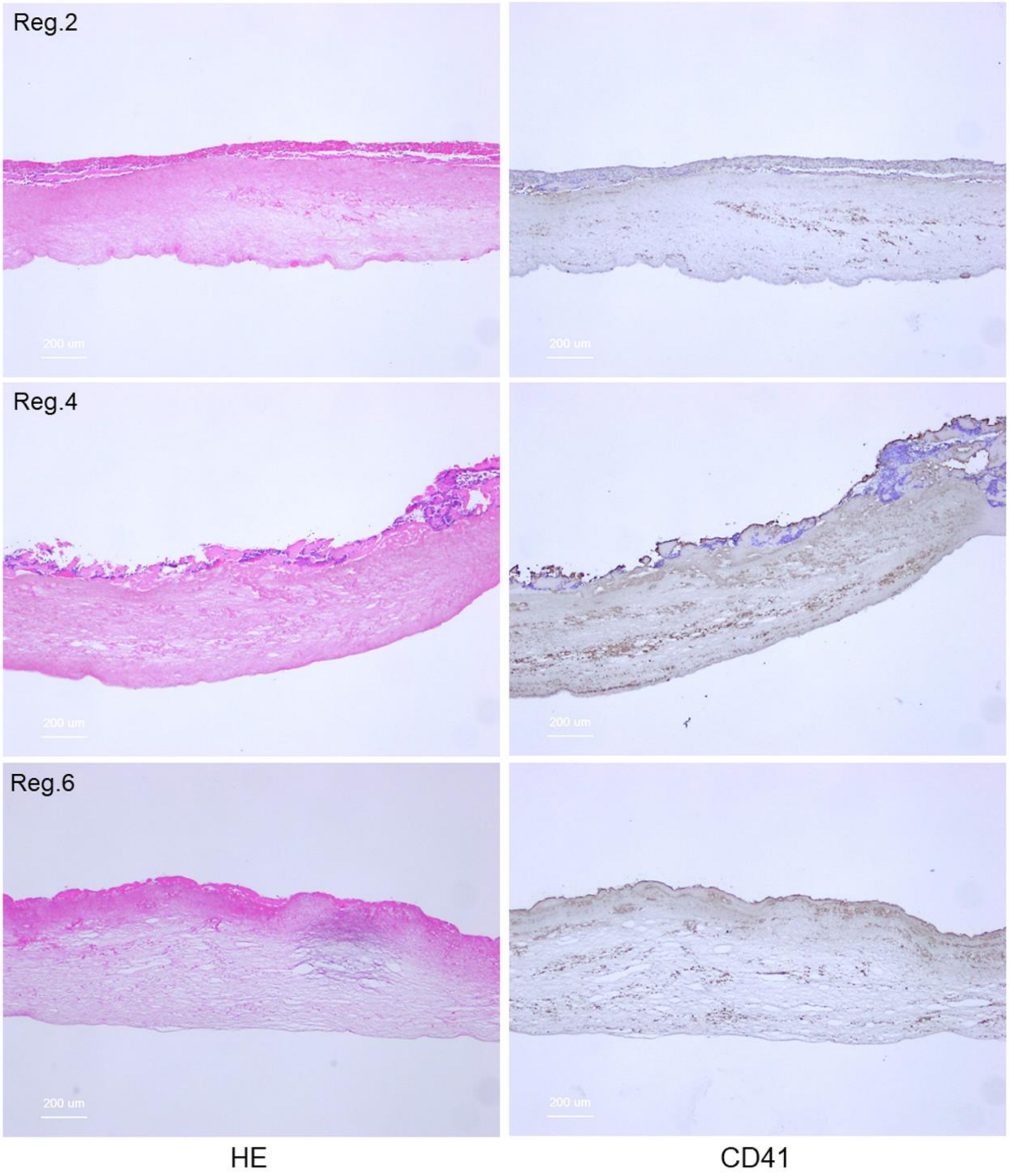

Figure S4. Platelet distribution in PRF matrix prepared using plastic, silica-coated tubes (Neotube) by low- (**a**: A-PRF protocol) and high-speed centrifugation (**b**: CGF protocol). Sections from regions (Reg.) 2, 4, and 6, stained with HE (left panels) or using anti-CD41 antibody (right panels) are shown. CD41⁺ platelets are stained dark brown.
